# Pleiotropic effects of *rfa*-gene mutations on *Escherichia coli* envelope properties

**DOI:** 10.1038/s41598-019-46100-3

**Published:** 2019-07-04

**Authors:** Christophe Pagnout, Bénédicte Sohm, Angélina Razafitianamaharavo, Céline Caillet, Marc Offroy, Marjorie Leduc, Héloïse Gendre, Stéphane Jomini, Audrey Beaussart, Pascale Bauda, Jérôme F. L. Duval

**Affiliations:** 1 Université de Lorraine, LIEC, UMR7360, Campus Bridoux, Metz, F-57070 France; 2 Université de Lorraine, LIEC, UMR7360, Vandoeuvre-lès-Nancy, F-54000 France; 3 Plateforme protéomique 3P5, Inserm U1016-Institut Cochin, Université Paris Descartes, MICUSPC, Paris, France; 4 ANSES, 94701 Maisons-Alfort Cedex, France

**Keywords:** Cell biology, Bacteriology, Biological physics

## Abstract

Mutations in the *rfa* operon leading to severely truncated lipopolysaccharide (LPS) structures are associated with pleiotropic effects on bacterial cells, which in turn generates a complex phenotype termed deep-rough. Literature reports distinct behavior of these mutants in terms of susceptibility to bacteriophages and to several antibacterial substances. There is so far a critical lack of understanding of such peculiar structure-reactivity relationships mainly due to a paucity of thorough biophysical and biochemical characterizations of the surfaces of these mutants. In the current study, the biophysicochemical features of the envelopes of *Escherichia coli* deep-rough mutants are identified from the molecular to the single cell and population levels using a suite of complementary techniques, namely microelectrophoresis, Atomic Force Microscopy (AFM) and Isobaric Tag for Relative and Absolute Quantitation (iTRAQ) for quantitative proteomics. Electrokinetic, nanomechanical and proteomic analyses evidence enhanced mutant membrane destabilization/permeability, and differentiated abundances of outer membrane proteins involved in the susceptibility phenotypes of LPS-truncated mutants towards bacteriophages, antimicrobial peptides and hydrophobic antibiotics. In particular, inner-core LPS altered mutants exhibit the most pronounced heterogeneity in the spatial distribution of their Young modulus and stiffness, which is symptomatic of deep damages on cell envelope likely to mediate phage infection process and antibiotic action.

## Introduction

Lipopolysaccharides (LPS) cover surface of the outer membrane of Gram-negative bacteria. They are tripartite molecules composed of lipid A, core oligosaccharides usually containing glucose, heptose, galactose, 2-keto-3-deoxyoctonate (KDO), and a highly variable O-antigen component (O-antigen is missing in *Escherichia coli* K-12). LPS act as a protective and permeable barrier against large molecules and hydrophobic compounds from the environment. They are positioned among phospholipids and proteins of the outer membrane, and contribute to the structural properties of the latter.

**Figure 1:**
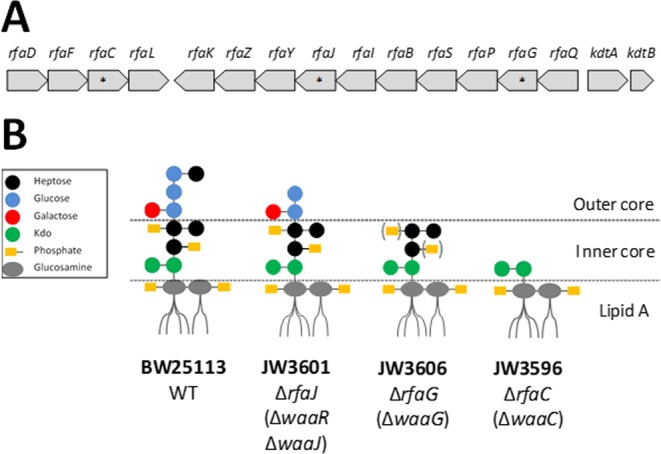
Schematic representation of the *rfa* operons in *E. coli* BW25113 (Wild Type) (**A**) and of the expected LPS structures of the strains used in this study (**B**). Positions of mutated genes in the *rfa* operon are indicated by (*). The brackets in the scheme of JW3606 indicate the 80% reduction in heptose phosphorylation. This figure is adapted from Yethon *et al*.^5^.

In *Escherichia coli*, genes involved in the LPS synthesis are organized according to three operons in the *rfa* (also known as *waa*) locus (Fig. 1A). The first operon contains *rfaD* (or *gmhD*), *rfaF*, *rfaC* and *rfaL* genes. The three first genes encode proteins involved in the biosynthesis and transfer of the two first heptose residues in the inner core of LPS, whereas *rfaL* encodes a ligase required for the attachment of O-antigen. The second operon contains (i) *rfaQ* and *rfaK* (or *waaU*) that encodes the heptosyltransferases adding the third and fourth heptose residues, respectively, (ii) genes *rfaG* (or *waaG*)*, rfaI* (or *waaO*) and *rfaJ* (or *warR* /*waaJ*) encoding the glucosyltransferases that add the three glucose residues in the LPS outer core, (iii) *rfaB* that encodes the galactosyltransferase adding the galactose residue to the first glucose, (iv) *rfaY* and *rfaP* that encodes kinases responsible for phosphorylation of heptoses, (v) *rfaZ* involved in the KDO attachment during LPS core biosynthesis, and finally (vi) *rfaS* that encodes a protein necessary for the attachment of rhamnose to the LPS core by linkage to the KDOII residue. The short *kdtA* operon contains *kdtA* (or *waaA*) that encodes the KDO transferase adding the two KDO residues to the lipid A and *kdtB* (or *coaD*) that is not involved in the LPS synthesis^1–3^. A defining feature of *E. coli* LPS is the presence of phosphoryl substituents on the LPS core-heptose residues, essential for membrane stability and structural cohesion. Due to their negative charges, these substituents enable divalent cations-mediated cross-linking of neighboring LPS molecule^4,5^.

Several studies have previously shown that mutation in the *rfa* locus leading to a severely truncated LPS in the core region, is often associated with a pleiotropic phenotype termed deep-rough^1,4,6^. The deep-rough phenotype includes a hypersensitivity to hydrophobic antibiotics (*i.e*. novobiocin, actinomycin D) and anionic detergents (*i.e*. sodium dodecyl sulfate)^1,4,7^, a drastic reduction of the outer membrane proteins, an increase of the phospholipids content, an enhanced resistance to several bacteriophages^1,8^, a production of colanic acid capsular polysaccharide^1^ and sometimes a loss of fimbriae and flagella expressions^9,10^ with major implications for their attachment to (a)biotic surfaces and their ability to form biofilms^3,10–12^. Recent studies based on large-scale phenotypic screening of the Keio collection (*i.e*. 3985 isogenic mutants of *E. coli* K-12) are consistent with these observations, thus highlighting a distinctive *rfa*-gene mutation phenotype compared to the wild-type strain with respect to *e.g*. susceptibility to bacteriophages^13–15^ and antibiotics^16,17^ and in terms of their ability to become non-motile^18^ or to form biofilms^19^. Despite the well-recognized specificities of deep-rough *E. coli* mutants, there is still an urgent need to achieve a comprehensive understanding and clear identification of the molecular determinants leading to their peculiar phenotypes. For that purpose, so far missing biochemical and physicochemical characterizations of the envelope of these mutants are mandatory.

In the current study, the pleiotropic effects associated with single *rfa*-gene mutations leading to inner (Δ*rfaC* and Δ*rfaG*) or outer (Δ*rfaJ*) core truncated LPS on *E. coli* K-12 membrane (Fig. 1B) are addressed by a suite of complementary experimental techniques probing cell envelope properties at different scales, from the molecular level, the single cell level up to the cell population, namely (i) iTRAQ-based quantitative proteomics for analysis of the cell envelope protein content, (ii) Atomic Force Microscopy (AFM) and Spectroscopy for cell imaging and evaluation of membrane elasticity and internal Turgor pressure (which is related to cell stiffness), and (iii) electrokinetics (electrophoretic mobility), which provides information on the bacterial surface charge density and on the permeability of the biosurface to the tangential electroosmotic flow developed during cell migration under action of an applied electric field. These data are thoroughly discussed in relation with the peculiar reactivity of deep-rough mutants of *E. coli* within the contexts of resistance/sensitivity to bacteriophages, antimicrobial peptides and hydrophobic antibiotics.

## Results and Discussion

### Electrokinetics

**Figure 2:**
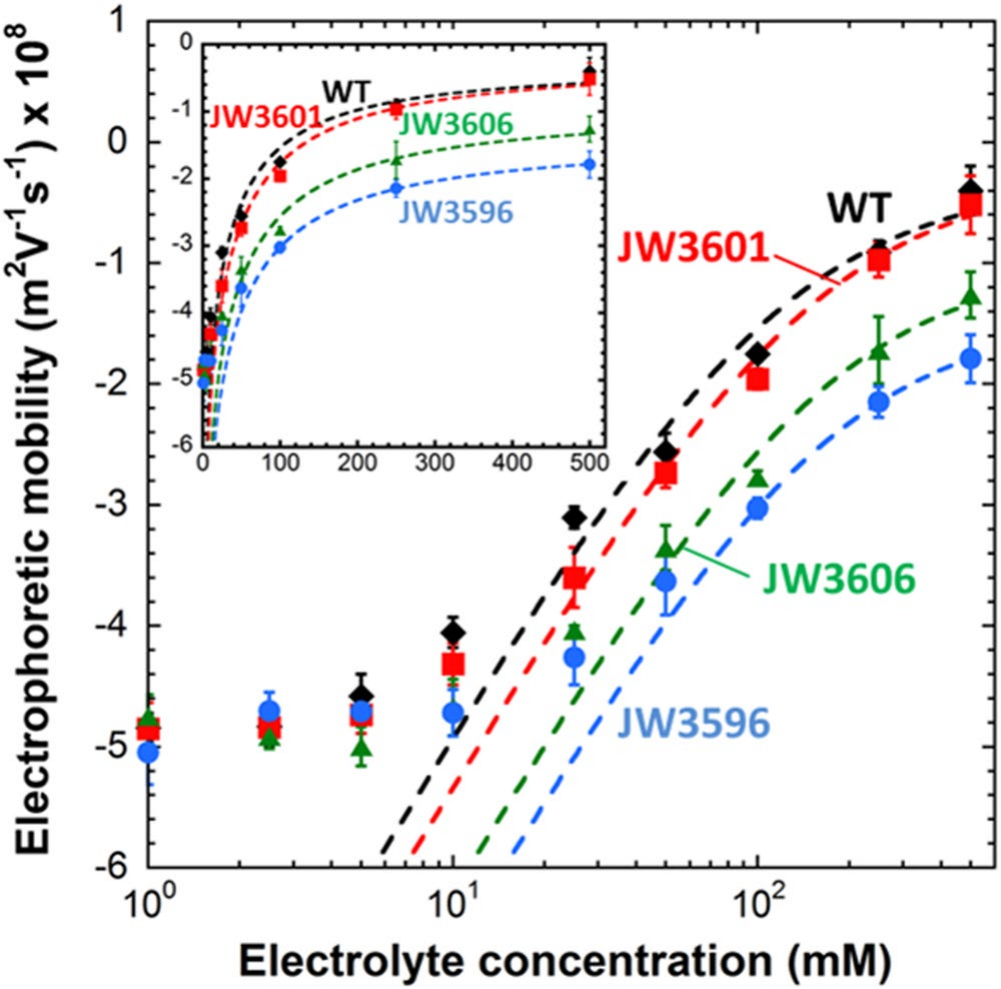
Dependence of the electrophoretic mobility on electrolyte concentration for the WT (black), JW3601 (red), JW3606 (green) and JW3596 (blue) cells. Points: experimental data. Dotted lines: theory (eqs 1–4). In inset, electrokinetic data are represented according to linear axis in electrolyte concentration.

Figure 2 reports the dependence of the electrophoretic mobility (*μ*) on KNO_3_ electrolyte concentration (denoted as *c*^∞^) for the wild type cells (WT) and for the knock-out mutants JW3601 (Δ*rfaJ*), JW3606 (Δ*rfaG*) and JW3596 (Δ*rfaC*) whose LPS compositions are pictured in Fig. 1. The mobility *μ* of the four strains of interest is negative over the whole range of electrolyte concentrations, which is in line with the protolytic properties of the functional groups carried by the outer membrane and the LPS structures. In addition, the absolute value of *μ* decreases with *c*^∞^ as a result of significant screening of cell surface charges by ions from the background electrolyte. The remarkable feature is the existence of a non-zero mobility plateau value at sufficiently large *c*^∞^ (inset Fig. 2), which is the characteristic electrokinetic signature for the presence of a *soft* surface layer (here the LPS-decorated membrane) surrounding the cells, *i.e*. a layer that is defined by a 3D distribution of ionogenic charges and that is further permeable to ions and to the electroosmotic flow developed during migration of the cells under the action of the applied electric field^20^. Accordingly, and in agreement with literature on electrokinetics of soft surfaces^21^ and of soft (bio)particles^20,22–24^, the conversion of electrophoretic mobility into zeta-potential value by means of *e.g*. the Smoluchowski equation or more sophisticated numerical theory^25^ is physically meaningless for such soft particles type. Indeed, contrary to their hard (*i.e*. ion- and flow-impermeable) counterparts, there is no way to unambiguously identify a slip plane and therewith define a zeta potential within their permeable peripheral polyelectrolyte-like envelope^26,27^. Instead, a quantitative rationale of the electrokinetic response of soft particles like bacteria requires resorting to theoretical framework that explicitly integrates the 3D distribution of charges within their surface envelope and their finite permeation to electroosmotic flow^20,22–24,26,27^. On a quantitative level, the electrokinetic measurements were collated here with the analytical theory on electrokinetics of soft (bio)particles as derived by Ohshima^22,23^. According to the latter, *μ* is provided by the expression^22,23^

1 $$\mu =\frac{{\rho }_{{\rm{o}}}}{\eta {\lambda }_{{\rm{o}}}^{{\rm{2}}}}+\frac{\varepsilon }{\eta }\frac{{\psi }^{{\rm{o}}}/{\kappa }_{{\rm{m}}}+{\psi }^{{\rm{D}}}/{\lambda }_{{\rm{o}}}}{1/{\kappa }_{{\rm{m}}}+1/{\lambda }_{{\rm{o}}}}$$

where ${\rho }_{{\rm{o}}}$ represents the effective volumic charge density in the cell soft surface layer, ${\kappa }_{{\rm{m}}}$ the reciprocal Debye thickness in that layer and ${\lambda }_{{\rm{o}}}$ the so-called softness parameter. The quantity $1/{\lambda }_{{\rm{o}}}$ corresponds to the characteristic penetration length of the electroosmotic flow within the outermost periphery of the soft surface structure. In the limit $1/{\lambda }_{{\rm{o}}}\to 0$, this peripheral region of the surface layer is impermeable to flow (situation met for so-called hard particles type) whereas the limit $1/{\lambda }_{{\rm{o}}}\to \infty$ denotes a surface layer that exerts no significant frictional forces on the flow (the limiting free-draining soft particle case^23^). In Eq. 1, ${\psi }^{{\rm{o}}}$ represents the surface potential, *i.e*. the potential at the position corresponding to the location of the outer boundary of the surface layer, and ${\psi }^{{\rm{D}}}$ the Donnan potential, *i.e*. the electrostatic potential reached within the bulk of that layer. The parameters ${\psi }^{{\rm{D}}}$, ${\psi }^{{\rm{o}}}$ and ${\kappa }_{{\rm{m}}}$ all depend on the space charge density ${\rho }_{{\rm{o}}}$ according to the following expressions (valid for a 1:1 background electrolyte)^22,23^

2 $${\psi }^{{\rm{D}}}=\frac{RT}{F}{\sinh }^{-1}(\frac{{\rho }_{{\rm{o}}}}{2F{c}^{\infty }}),$$

3 $${\psi }^{{\rm{o}}}={\psi }^{{\rm{D}}}-\frac{RT}{F}\,\tanh (\frac{F{\psi }^{{\rm{D}}}}{2RT}),$$

4 $${\kappa }_{{\rm{m}}}=\kappa {\{\cosh (\frac{F{\psi }^{{\rm{D}}}}{RT})\}}^{1/2}$$

where *R* is the gas constant, *T* the absolute temperature, *F* the Faraday number and $\kappa$ the reciprocal Debye layer thickness operational at the solution side of the cell/solution interface. The fitting of the cell mobility *versus* electrolyte concentration curves displayed in Fig. 2 was obtained after adjustment of the sought parameters ${\lambda }_{{\rm{o}}}$ and ${\rho }_{{\rm{o}}}$ according to least-squares method. As extensively detailed in previous reports^20^, reproduction of experimental data is excellent at sufficiently large electrolyte concentrations (here above *ca*. 25 mM) where the assumptions underlying the validity of Eqs 1–4 are verified, namely: Donnan electrostatics applies within the soft surface layer, double layer polarization by the applied electric field is negligible, and the magnitude of the interfacial potentials is sufficiently low (*ca*. <40–50 mV) to warrant application of the Debye-Hückel approximation. For the cells harvested according to the protocol detailed in the experimental section, the obtained flow penetration length is $1/{\lambda }_{{\rm{o}}}$ = 0.61 ± 0.07 nm for JW3606 and JW3596, and $1/{\lambda }_{{\rm{o}}}$ = 0.29 ± 0.04 nm for JW3601 strains and WT cells, respectively. These values all correspond to the low limit of the spectrum of $1/{\lambda }_{{\rm{o}}}$ values inventoried by Duval and Gaboriaud^20^ for various Gram-negative and Gram-positive bacteria. They are symptomatic of moderately permeable bacterial envelops and of outer membranes devoid of thick and loose biopolymeric surface structures. In addition, the (significantly) larger $1/{\lambda }_{{\rm{o}}}$ values obtained for JW3606 and JW3596 (*i.e*. the strains with inner core truncated LPS) may be the result of their increased membrane destabilization following the reduction in the negative charges caused by the loss of phosphoryl groups^5^. Such destabilization process is indeed probably accompanied by an increasing membrane permeability, which is reflected here by a significant increase of $1/{\lambda }_{{\rm{o}}}$ that basically leads to higher magnitude of the mobility plateau reached at large electrolyte concentrations for JW3606 and JW3596. Previous results indicated that the suppression of flexible bacterial membrane-supported soft surface appendages is accompanied by a decrease in $1/{\lambda }_{{\rm{o}}}$ on the premise that membrane permeability remains unchanged^24^, a finding that is not observed here upon LPS truncation. The charge density of the membrane-supported surface layer as derived from Eqs 1–4 (and expressed in equivalent concentration of monovalent anionic charges) is 305±15 mM for JW3606, JW3596 and JW3601 strains, and 242 ± 10 mM for WT cells, respectively, all being qualitatively in line with the range of ${\rho }_{{\rm{o}}}$ values detailed by Duval and Gaboriaud^19^ for bacteria characterized by sub-nanometric to nanometric flow penetration length scales. The obtained magnitude of ${\rho }_{{\rm{o}}}$ highlights that knock-out mutants and WT cells carry here relatively similar *density of charges* at their outer periphery. Indeed, recalling that ${\rho }_{{\rm{o}}}$ mostly determines *the rate of increase* of the cell mobility (in absolute value) with decreasing the electrolyte concentration, the shape of the electrokinetic profiles of the 4 cell strains in Fig. 2 basically remains identical (and, therefore, so does ${\rho }_{{\rm{o}}}$) at sufficiently large electrolyte concentrations where Eqs 1–4 are strictly applicable. The only major difference originates from their respective shift upwards or downwards, the latter shift being determined by the magnitude of $1/{\lambda }_{{\rm{o}}}$. The inherent scattering of the data at large electrolyte concentrations prevents from drawing a refined quantitative appreciation of the *respective* electrostatic features (*i.e*. ${\rho }_{{\rm{o}}}$) of the strains investigated. In turn, the decrease of the amount of charges within the LPS layer upon truncation and the concomitant reduction of the surface layer volume where the remaining charges are distributed seems to lead to nearly unchanged charge density for the *rfa*-gene mutants and -to a somewhat lesser extent- for the WT. Obviously, further LPS surface characterization at the single cell level is required to (i) better discriminate the action of LPS truncation on membrane destabilization for the various cell strains, and (ii) refine the (cell population-averaged) information derived above from electrokinetic measurements. To meet this requirement, cell imaging and nanomechanical properties as derived from AFM are detailed in the next section.

### AFM analysis of cell surface properties

**Figure 3:**
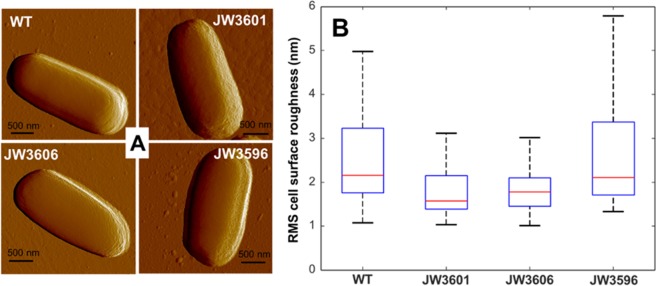
Representative peak force error images of WT, JW3601 (Δ*rfaJ*), JW3606 (Δ*rfaG*) and JW3596 (Δ*rfaC*) cells (**A**) and RMS cell surface roughness (*R*_surface_) for the four strains of interest in this work (**B**). In order to evaluate the statistical dispersion of *R*_surface_, we use a classical whisker representation for which the bottom and the top of the box are the 25th and the 75th percentiles noted, respectively, 25Q and 75Q. The bold red band in the box corresponds to the median. The ends of the whiskers represent (i) the largest measured cell surface roughness that is less than or equal to the third quartile plus 1.5 times the interquartile range (75Q-25Q), and (ii) the lowest measured cell surface roughness that is larger than or equal to the first quartile minus 1.5 times the interquartile range (75Q-25Q). Data in panel B stem from measurements conducted on 3 to 12 different bacteria (probed cell surface area 500 × 500 nm^2^) per cell culture and 7 to 11 different cell cultures. Overall, *ca*. 50 cell images per strain were considered for evaluation of RMS cell surface roughness.

Figure 3A displays illustrative peak force error images obtained for JW3606 (Δ*rfaG*), JW3596 (Δ*rfaC*), JW3601 (Δ*rfaJ*) and WT cells. Overall, cell surfaces do not exhibit extended protruding surface appendages and appear ‘rather smooth’ without marked asperities or protrusions. Figure 3A shows that there is no difference in terms of morphology or of other microscale-surface features among the 4 bacterial strains investigated in this study. The flexibility of the expected few nanometers-long LPS structures (or even longer structures of the flagella type if present) renders their detection impossible by AFM under liquid conditions, a conclusion in line with previous reports^24^. Refined analysis reveals that the root mean square (RMS) surface roughness (denoted as *R*_surface_) as evaluated for WT, JW3601 and JW3606 decreases and subsequently levels off (on a statistical basis, see bottom and top limits of the box-plot representation provided in Fig. 3B) with the extent of LPS truncation, and *R*_surface_ of JW3606 is significantly lower than that for JW3596 which exhibits the shortest LPS structure (*R*_surface_ = 1.8–3.2 nm, 1.4–2.2 nm, 1.5–2.1 nm and 1.7–3.4 nm for WT, JW3601, JW3606 and JW3596 cells respectively where the first and second number correspond to the 25th and the 75th quartiles, respectively, see Fig. 3B for details). This intricate parabolic-like evolution of *R*_surface_ originates from both a cell surface smoothing that follows truncation of the protruding LPS chains, *and* from associated irregularities of the supporting outer cell membrane surface (roughing process). The respective *R*_surface_ obtained for WT and JW3601 indicate that LPS truncation basically ‘flattens’ the cell surface, as intuitively anticipated. However, the significantly larger range of *R*_surface_ values derived for JW3596 further suggests that a nearly complete suppression of LPS structure induces noticeable asperities/depression at the outer membrane underneath. The situation for JW3606 is intermediate between these two extremes, thereby resulting in a shallow minimum for *R*_surface_ as a function of the LPS truncation degree. This result qualitatively corroborates the effects of LPS truncation on membrane integrity (and resulting developments of cell surface irregularities), as revealed by electrokinetics *via* the hydrodynamic penetration length scale $1/{\lambda }_{{\rm{o}}}$ that is *ca*. two times higher for JW3596/JW3606 as compared to that derived for WT/JW3601 cells. It is emphasized that results in Fig. 3B are derived from AFM images performed on a ‘limited’ number of cells (*ca*. 50), a feature that is inherent to the use of AFM-based analyses of single cells. The statistics of the data was established from measurements performed on a cells number that is however relatively large as compared to that commonly reported in various AFM studies on biological cells (where *ca*. 10–20 cells are examined)^28^.

**Figure 4:**
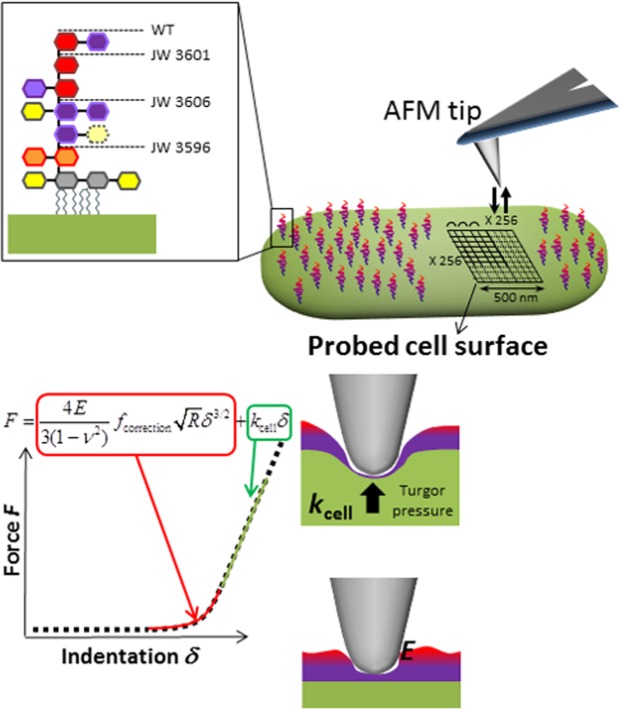
(Top panel). Schematic representation of nanomechanical/indentation force measurements between an AFM tip and the bacterial cell surfaces investigated in this study. (Bottom panel). Illustration of the procedure according to which internal Turgor pressure (or, equivalently, cell spring constant also termed cell stiffness, *k*_cell_) and Young modulus (*E*) of the cell envelope are retrieved from analysis of force (in N)-indentation (in nm) curves. The equation refers to the nanomechanical model adopted, with a non-linear Hertz-Dimitriadis contribution (red) and a linear compliance component (green). ν is the Poisson ratio (=1/2), *f*_correction_ is the factor elaborated by Dimitriadis *et al*.^72^ that corrects the Hertz model for finite sample thickness (here the height of a bacterium, *ca*. 800 nm), and *R* (=20 nm) corresponds to the radius of the hemispherical tip apex. Under the conditions examined in this work, the non-linear indentation domain extends over *ca.* 20 to 70 nm inside the peripheral cell envelope.

**Figure 5:**
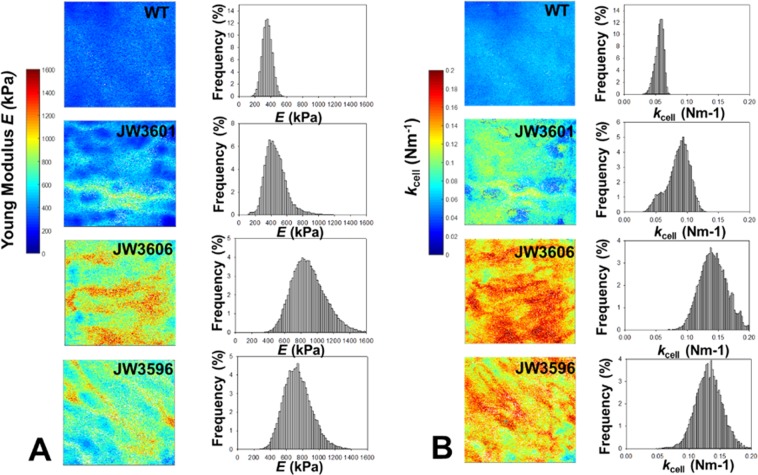
(**A**) Illustrative Young modulus (*E* in kPa) distribution over a 500 × 500 nm^2^ (256 × 256 force curves) scanned cell surface area (left column) of WT, JW3601, JW3606 and JW3596 cells (indicated) and corresponding spatial frequency distributions of *E* (right column). (**B**) As in panel A for the cell spring constant (*k*_cell_ in Nm^−1^). Young moduli and cell spring constants were evaluated from theoretical fitting of the force *vs*. indentation curves collected at various locations of the cell surface, as illustrated in Fig. 4.

**Figure 6:**
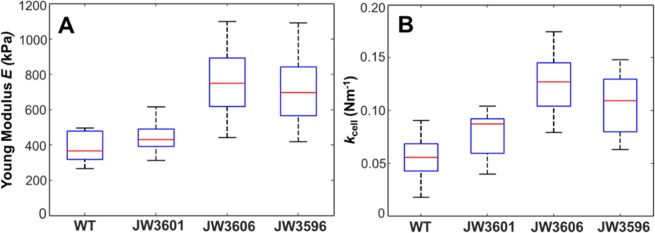
Dependence of the Young modulus (**A**) and cell spring constant (**B**) on the cell strain considered. The meaning of the box plot is identical to that detailed in Fig. 3B for the RMS cell surface roughness. Data stem from measurements conducted on 2 to 3 different bacteria per cell culture and 5 to 6 different cell cultures. Overall, *ca*. 15 cell images per strain were considered for evaluation of *E* and *k*_cell_.

Following the strategy schematically summarized in Fig. 4, illustrative spatial distributions of Young modulus *E* and cell Turgor pressure (expressed in terms of an equivalent cell spring constant also called cell stiffness, denoted as *k*_cell_) are reported in Fig. 5 (*E* is provided in panel A-right column and *k*_cell_ in panel B-right column) and were derived from force measurements over a 500 × 500 nm^2^ scanned cell surface area (panels A & B-left columns). For the sake of statistical representation, the range of Young modulus *E* and *k*_cell_ derived over the ensemble of the measurements performed on different cells from the same or different suspensions (see the Experimental Procedure section), are given in the form of ‘box-plots’ in Fig. 6. The ensemble of the results displayed in Figs 5, 6 evidences that the inner core truncated mutants (JW3606 and JW3596) are significantly more rigid (*i.e*. they display higher modulus *E* and higher cell stiffness *k*_cell_) than the WT and the outer core truncated JW3601 mutant (Fig. 6), and that the spatial distribution of the nanomechanical *E* and *k*_cell_ characteristics defining JW3606 and JW3596 cells is more heterogeneous (patchy-like) than that pertaining to WT and JW3601 cells (Fig. 5).

These results indicate that the presence of LPS and their truncation significantly impact on cell deformation upon compression by the AFM tip (*i.e*. they affect *E*). LPS surface structures contribute to softening of the overall cell envelope (*i.e*. decreasing *E*) and the more so the thicker the LPS layer (Fig. 6). Obviously, for sufficiently short LPS structures, the effective Young modulus of the cell envelope (consisting of the LPS and supporting outer membrane) should basically reflect -in an asymptotic manner- the only contribution of the outer membrane, which is in line with the data of Fig. 6. The presence of an extended biopolymeric cushion on the outer membrane thus enhances the overall mechanical softness of the cell envelope, which is qualitatively in agreement with the results described elsewhere^24^ obtained for various *E. coli* K-12 mutants decorated by short-rigid adhesins or long-flexible type 1 *fimbriae* and F *pilus*. Within the compliance regime reached at sufficiently large tip indentations within the cell envelope (Fig. 4), compression of the latter becomes counteracted by intracellular force that originates from the Turgor pressure expressed here in terms of *k*_cell_. Figure 6 highlights that the evolution of *k*_cell_ from one cell strain to another closely follows that obtained for the Young modulus. This finding is explained by the dependence of the membrane stretching modulus and of the cell surface energy on the presence/absence and type of flexible surface structures at the bacterial envelope, as discussed elsewhere^24^.

In line with electrokinetics and cell surface roughness analyses (Figs 2 and 3B, respectively), the respective nanomechanical features of the cells of interest support that LPS truncation impacts on membrane cohesion and permeability. In particular, this effect is reflected by the bell-shape dependence of *E* and *k*_cell_ on LPS truncation degree, more exactly by the presence of a shallow maximum in *E* and *k*_cell_ (Fig. 6). This maximum originates from two opposite LPS truncation-mediated impacts on overall cell envelope mechanics: increasing rigidity (*E*) of the cells envelope upon moderate LPS truncation, as argued above, and decreasing rigidity for destabilized membranes following significant LPS truncation and associated reduction of negative charges caused by the loss of phosphoryl groups^5^. For JW3606 (maximum in *E* and *k*_cell_, Fig. 6), it is likely that both processes are significant and that they counterbalance each other, whereas the Young modulus of JW3596 with shortest LPS (point after the maximum in Fig. 6) is more dominated by membrane destabilization process, and that of JW3601 (point before the maximum in Fig. 6) is significantly impacted by cell envelope softening due to LPS-truncation (as compared to the WT reference situation). It is remarkable that the most pronounced heterogeneous distributions of *E* and *k*_cell_ are observed over the JW3606 mutant surface that exhibits the largest *E* and *k*_cell_. We believe that the joint membrane destabilization and significant LPS truncation (that lead to decreasing and increasing cell envelope rigidity if considered independently, respectively) results in the heterogeneous patterns displayed in Fig. 5, especially for JW3606 and, to a smaller extent, JW3596. Interestingly, the spatial domains identified in Fig. 5 from the *E*-maps do not necessarily coincide with those revealed by the *k*_cell_-maps. This feature is consistent with the above representation, recalling that *E* particularly pertains to the *outer* cell surface zone and *k*_cell_ mostly reflects integrity of the *whole* membrane region (see Fig. 4). The location of the partial to severe damages possibly caused by LPS truncation at the level of the membrane underneath (pleiotropic effects), which mostly affects *k*_cell_, may not necessarily coincide with the position of the LPS, the latter predominantly impacting the magnitude and spatial distribution of *E*.

In the section below, the preceding analyses of the electrokinetic, roughness and nanomechanical features of the 4 strains of interest in this work are completed by an iTRAQ-based quantitative proteomic study.

### Quantitative evaluation and identification of membrane proteins

**Figure 7:**
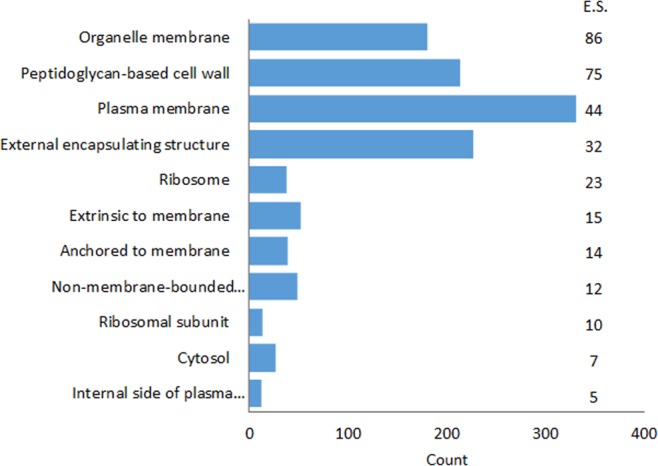
GO term (Cellular Component) enrichment analysis for proteins isolated from *E. coli* strains (WT and knock-out mutants). E.S. means ‘Enrichment Score’.

Membrane proteins were isolated by carbonate extraction as detailed in the experimental section. Incubation of cell membrane preparations with sodium carbonate solution enriches cell membrane proteins by stripping away other protein contaminants loosely associated to the membranes^29,30^. Extractions performed on a fixed number of cells (10^12^ cells as adjusted by flow cytometry counting) allow the collection of 1260 µg, 1610 µg, 1430 µg and 1610 µg of proteins from WT, JW3601 (Δ*rfaJ*), JW3606 (Δ*rfaG*), and JW3596 (Δ*rfaC*), respectively. ITRAQ analysis was performed on an equal amount of membrane proteins for each sample, which is required for a proper comparative study. A total of 645 proteins was identified (≥2 unique peptides assigned with high confidence (>95%)). By means of DAVID analysis, 429 proteins were classified into the Cellular Component GO term. As expected on the basis of the extraction procedure, the analysis revealed that most of the isolated proteins are located in the cell envelope, with a majority affiliated to the plasma membrane (331 proteins) (Fig. 7).

**Figure 8:**
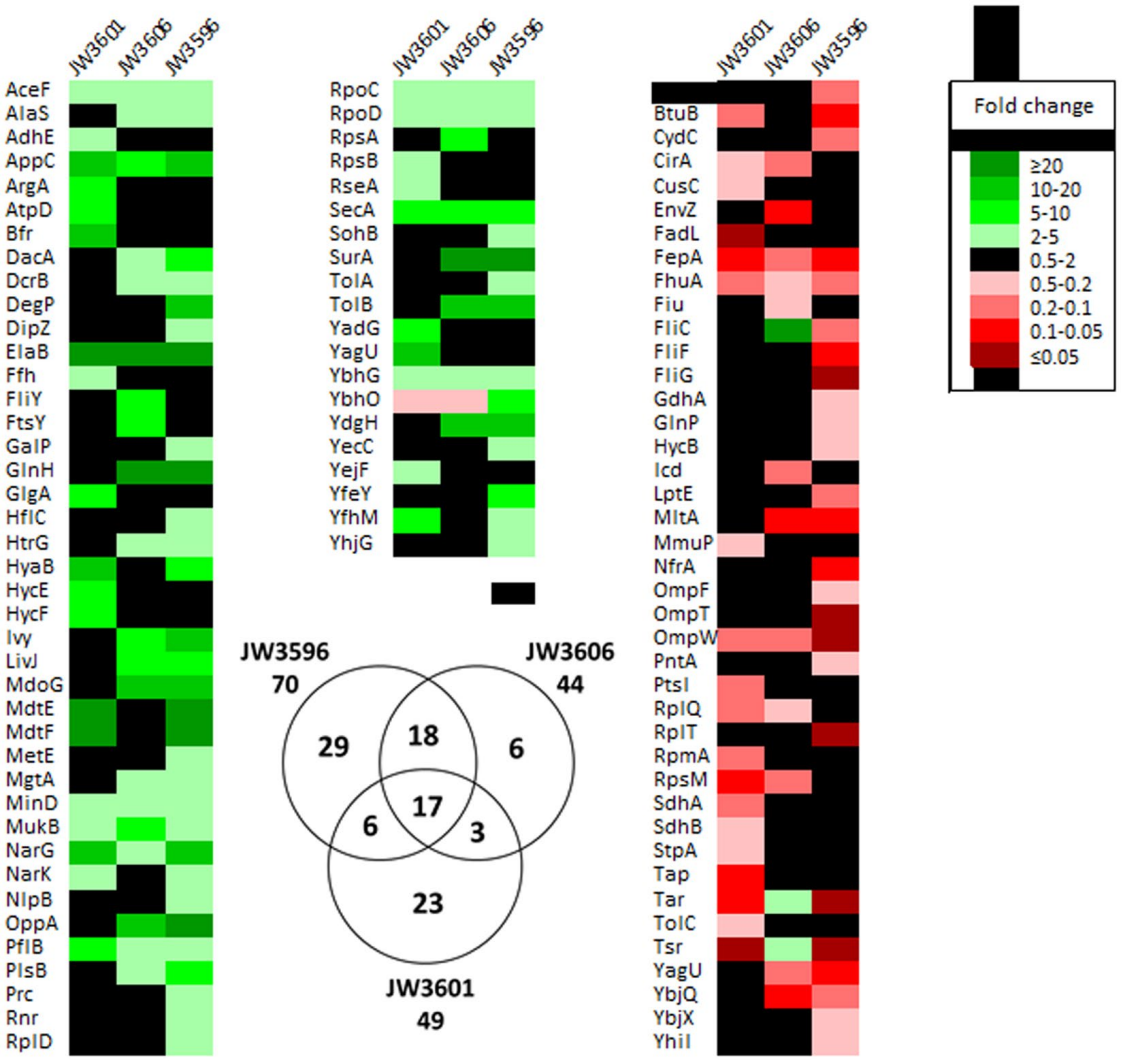
Heat map view generated from the 645 proteins identified from the iTRAQ analysis showing their relative abundance in *rfa* mutants compared to the WT reference strain. The increased and decreased abundance in proteins are indicated by the range of green and red color intensities, respectively. Only proteins deregulated in at least one of the three mutants are listed. The Venn diagram indicates the number of differentially abundant proteins shared by the three mutants.

Starting with this pool of 645 identified proteins, quantitative proteomic analysis based on the iTRAQ ratios reveals differential abundance (fold-changes ≥2 or ≤0.5 and 3 *p-*values ≤ 0.05) of 70 proteins in JW3596 (*rfaC* mutation), 44 proteins in JW3606 (*rfaG* mutation) and 49 proteins in JW3601 (*rfaJ* mutation) as compared to the WT reference strain. Among these proteins, 35 are common between JW3596 and JW3606, 20 between JW3606 and JW3601, and 23 between JW3601 and JW3596, while 17 proteins are common to the three *rfa* knock-out mutants (Fig. 8). A detailed list of these 17 proteins and of their functions is given in Supplementary Information, Fig. S1. Their possible relationship with the deep-rough phenotype is further discussed in the next section. The lists of the iTRAQ ratios, protein scores and accession numbers, percentages of sequence coverage and numbers of identified peptides are provided for each protein in Supplementary Data (Supplementary Table S1). We further report in Supplementary Fig. S2 the Western blot experiments for iTRAQ validation.

**Figure 9:**
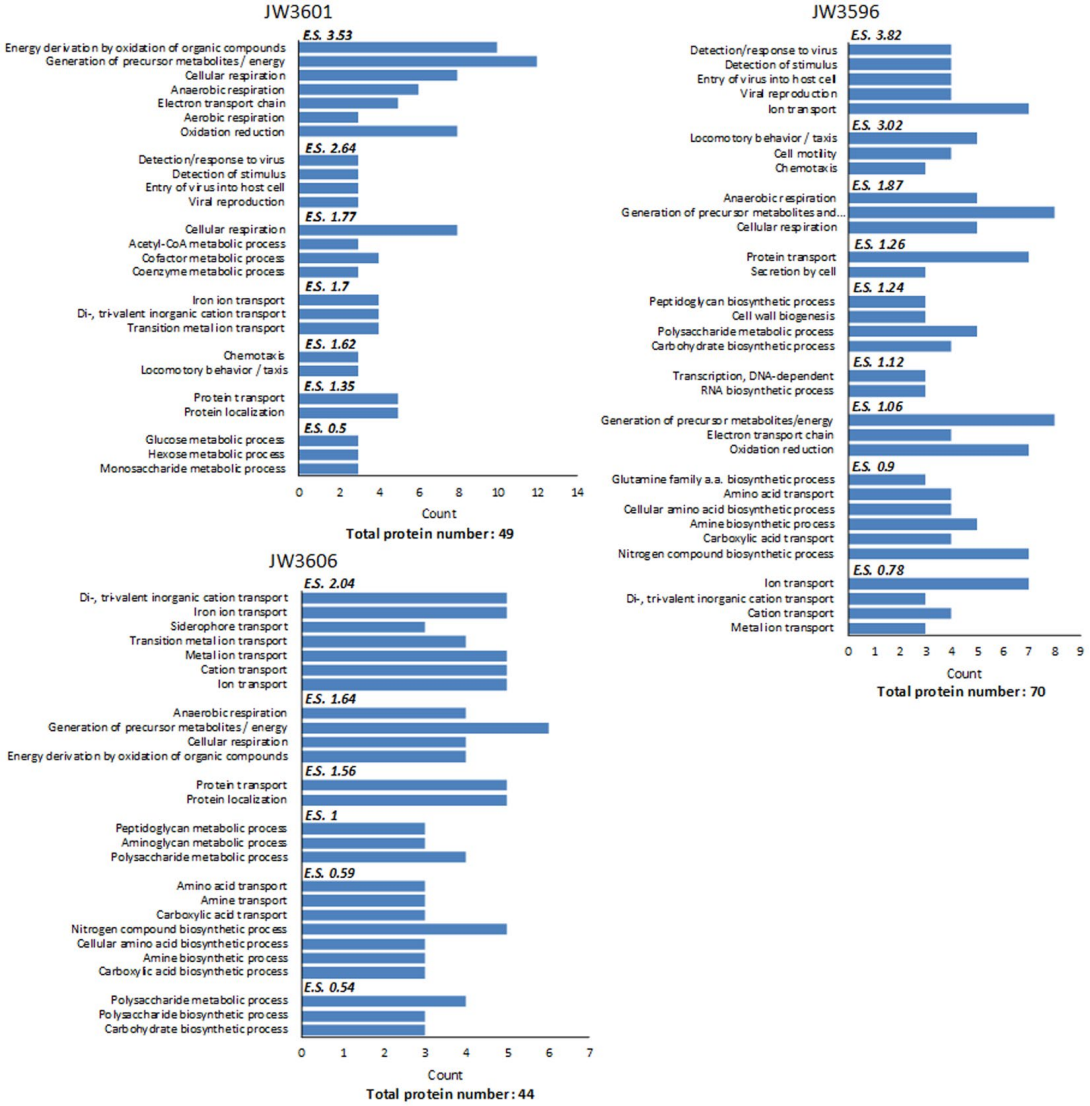
GO term (Biological Process) enrichment analysis for differentially abundant proteins identified by iTRAQ analysis. *E.S*.: Enrichment Score of different clusters.

For each strain, the list of differentially abundant proteins was subjected to functional annotation clustering using DAVID analysis. Data treatment highlights that the most enriched clusters (score > 0.5) are related to detection/response to viruses (JW3596 and JW3601), chemotaxis and locomotary behavior (JW3596 and JW3601), cellular respiration and energy derivation by oxidation of organic compounds (JW3596, JW3601, JW3606) and by transportation of amino acids/carboxylic acids (JW3596 and JW3606), of proteins (JW3596, JW3601, JW3606) and ions (JW3596, JW3601, JW3606) **(**Fig. 9).

### Pleiotropic effects in deep-rough mutants: a track to understand their peculiar phenotypes?

Deep-rough mutants were previously characterized by a significant (50 to 60%) reduction of their outer membrane protein (OMP) content as compared to that in WT reference^1,4,6^. Whereas no consensus has been reached on the origin of such a strong reduction, explanation attempts invoke an impaired ability of knock-out mutants to form stabilized porin trimers^8^, or a loss of improperly anchored proteins at the interface between cell and external aqueous medium^31^. Regardless of the possible scenario in line with OMPs reduction, the void left by the missing OMPs is filled by phospholipids, which leads to the occurrence of a phospholipid bilayer organized in patches at the outer membrane^4^ and consequently to higher cell permeability to hydrophobic compounds^32^. In the current study, the abundance of cell envelope proteins is shown to be dependent on the *rfa* gene mutation (Fig. 8). However, the dramatic decrease (more than 90%) of porins OmpA, OmpF and OmpC argued by several authors^1,4,9,32^ is not supported by our experimental data (Fig. 8 and Fig. S3). On the opposite, the results detailed here are fully consistent with those obtained by Yethon *et al*. who showed that specific deletion of *rfaP* and *rfaG* did not lead to any decrease in OmpA, OmpC, or OmpF contents, despite the hypersensitivity to novobiocin and sodium dodecyl sulfate (SDS), features that are characteristic of deep-rough phenotypes^5^. Yethon *et al*. argued that the discrepancy between their results and the existing literature could be due to mutations left uncharacterized in earlier mutants, or to the impacts of differences in genetic backgrounds on literature data^5^. From our point of view, it is the deletion strategy used to construct the knock-out mutants and the thereby-generated polar effects that are the central issue. Indeed, in order to avoid translational polarity, Yethon *et al*. used an insertion cassette (aacC1) that did not contain transcriptional stop for their mutant constructions^5^. The Keio *rfa*-mutants used in the current study were constructed by a homologous recombination mediated by the λ phage Red proteins, also termed recombineering strategy^33^. Following such a methodology, genes are targeted individually and mutants are nonpolar because genes are expressed from the resistance gene promoter or from the upstream native promoter pending elimination of the resistance cassette with use of the FLP recombinase^33^. In contrast, in previous studies of deep-rough mutants, authors adopted random insertion mutagenesis of transposons, which causes most of the time an incomplete disruption of the targeted gene and polar effects on the downstream gene expression^33^. It appears essential to consider this issue when interpreting the phenotype of a transposon insertion mutant, having in mind that the phenotype may then be due to polarity effects on a downstream gene and not directly to the inactivation of the gene that contains the insertion.

Additional complementation experiments performed on the Keio *rfa*-mutants investigated in this work unambiguously confirm that their associated deep-rough phenotype is specifically due to inactivated genes (Supplementary Information, SI-5). Indeed, it is established here that the strains JW3601, JW3606 and JW3596 carrying the plasmids pCA24N::*rfaJ*, pCA24N::*rfaG*, pCA24N::*rfaC*, respectively, exhibit a phenotype that is similar to that of the strain BW25113 (WT) transformed with the empty plasmid pCA24N. This conclusion is further supported by Minimum Inhibitory Concentration (MIC) experiments with SDS (Supplementary Table S2), and by AFM analyses that reveal similar surface roughness (Supplementary Fig. S4A) and similar nanomechanical properties (Supplementary Fig. S4B,C) for JW3601 pCA24N::*rfaJ*, JW3606 pCA24N::*rfaG*, JW3596 pCA24N::*rfaC* and for the reference strain BW25113 containing the empty plasmid pCA2AN. The reader is referred to SI-5 for further details.

Since 2006, date on which the *Escherichia coli* Keio collection 3985 mutants was made available to the scientific community, numerous studies have screened and identified several *rfa*-gene mutants for their peculiar susceptibility to various bacteriophages and antibacterial substances (*e.g*. antibiotics, bacteriocins and detergents). However, due to a lack of characterization of these mutants, there is a remaining controversy on the nature of the biomolecular determinants at the origin of the newly observed phenotypes. Within this context, the data reported in this work bring new elements for a comprehensive understanding of the peculiar deep-rough phenotypes, as detailed in the following sections.

#### Resistance to bacteriophage

Historically, deep-rough mutants are known to be resistant to several bacteriophages^1,15^, in line with recent screenings of the *E. coli* Keio mutants collection based on phage resistance detection^13–15^. For the sake of illustration, Qimron *et al*. identified 11 mutants resistant to phage T7 from the 3985 tested mutants^13^. Among them, 9 mutants with altered LPS biosynthesis were isolated. Following a similar strategy, Maynard *et al*. identified 57 mutants, including 8 *rfa*-mutants, with reduced infectivity to phage lambda^14^. It is *a priori* well accepted that LPS recognition by the phage receptor is essential for phage adsorption to Gram-negative host cell. However, some studies suggested that LPS play a non-determining role in the process leading to cell infection by phage^15,34^ and that the sole LPS truncation cannot explain the significant levels of resistance observed for deep-rough mutants. Starting from this observation, several authors considered the possible reduction of OMPs, also commonly acting as receptors for phages^35^, as the origin of enhanced resistance to phage infection^15,36^. However, to the best of our knowledge, this hypothesis has never been clearly validated. It is here elegantly supported by our iTRAQ data and enrichment analysis procedure as reported in Figs 8 and 9. Indeed, mutants JW3596 and JW3601 show an enrichment score of 3.82 and 2.64 on GO term *detection/response to virus*, which corresponds, for JW3596, to 4 OMPs (FhuA, BtuB, OmpF, NrfA) among the 70 differentially abundant proteins and, for JW3601, to 3 OMPs (FhuA, BtuB, FadL) among the 49 we identified (Fig. 9). Strain JW3606 does not pass the minimum enrichment score for this term as only 1 OMP (FhuA) matches among the 44 differentially abundant proteins. Protein FhuA (also called TonA), that is 2.4 times less abundant in JW3606 strain and more than 6 times less present in JW3596 and JW3601 compared to WT, is the receptor of T7, T5, T1, ϕ80, UC-1 and many other *E. coli* phages isolated from nature^37^. In the study by Qimron *et al*., the three deep-rough mutants termed *waaC*, *waaR* and *waaG* (which corresponds to our JW3596, JW3601 and JW3606, respectively) were found resistant to phage T7^13^. They also highlighted that mutants JW3596 and JW3601 have a level of resistance higher than that of the JW3606 mutant, which is in good agreement with the lower content of the FhuA-phage receptor evidenced by our data. In the same way, Trojet et *al*. tested efficiencies of T4 Superfamily Phages on *E. coli* Keio strains with single deletion of genes coding for known phage receptors^38^. They found very similar resistance profiles for JW3606 (Δ*rfaG*) and JW0146 (Δ*fhuA*) to PST, SV76, RB51 and RB69 phages in agreement with similar level of FhuA abundance. However, due to a lack of literature data, such correlations could not be established with BtuB, NrfA, FadL and OmpF, which are protein receptors of the bacteriophages BF23^39^, N4^40^, T2^41^ and K20^42^. The nanomechanical properties of the knock-out mutants as compared to those pertaining to WT (Figs 5 and 6) are also in line with enhanced resistance of JW3596, JW3601 and JW3606 to phage infection. Indeed, the larger Young modulus measured for the envelopes of these mutants and their larger inner turgor pressures likely limits any *deep* perforation of the whole cell envelope and/or ejection of DNA from tailed phage into bacteria cytosol compartment during the infection process, and thereby contribute to increase resistance pattern. The predominant role of internal cell turgor pressure in phage DNA ejection was previously demonstrated by several authors^43–45^. Finally, the analysis of the respective electrokinetic responses of WT and of the mutants reveals that their common electrostatic surface properties cannot explain any differentiated capacities to bind (negatively charged) viruses and, in turn, cannot account for the reported enhanced resistance of the mutants compared to WT cells. These elements illustrate the different implications of gene mutations not only in terms of OMP composition, but also in terms of change in the nanomechanical properties of the cell envelopes.

#### Resistance to cationic antimicrobial peptides

Deep-rough mutants show higher sensitivity to many antimicrobial peptides (AMPs) compared to the parental strain^46,47^. Recent analysis of the susceptibility of several Keio *rfa*-mutants toward the α-helical peptides Cap18, Cap11-1-18m^2^, Cecropin P1, Cecropin B, Indolicidin, Melittin and Sub5 revealed an enhanced sensitivity of JW3596 (Δ*rfaC*) and JW3606 (Δ*rfaG*) compared to WT cells (strain JW3601 (Δ*rfaJ*) was not tested in this work)^47^. This feature can be explained by the respective protrusions of their LPS surface layer (Fig. 1) acting as a more or less efficient protective (steric) barrier against harmful AMP effects. In addition, via electrostatic bonding, cationic AMPs may attach to and insert into the anionic cell surface to form transmembrane pores whose accessibility for AMPs is mediated by the dimension of the peripheral LPS layer^48,49^. Our electrokinetic data (Fig. 2) suggest however that electrostatic interactions between negatively-charged cells surface and positively charged AMPs should not be significantly different for WT cells and *rfa*-gene mutants (whose soft peripheral surface layers are defined by similar densities of electrostatic charges), which in turn would indicate that AMPs action is not predominantly governed by cell surface electrostatics. This finding is in line with the conclusions by Jacquet *et al*. who evidenced the absence of correlation between physicochemical cell surface properties and activity of small AMPs of the type carnobacteriocins^50^. The sensitivity of JW3606 and JW3596 to active AMPs (see examples above) is further likely favored by their pronounced membrane destabilization and enhanced peripheral cell permeability as evidenced from electrokinetics (via evaluation of the hydrodynamic softness parameter) and nanomechanical cell analysis (see Fig. 5 and the marked patchy distributions of *E* and *k*_cell_ illustrated therein for the inner core truncated LPS displayed by the JW3606 and JW3596 strains).

Further recent studies have shown that susceptibility to bactericidal effects of AMPs is not universally mediated by LPS structure but also by outer membrane proteins^51–53^. These proteins may operate as entry or excretion channels for AMPs and can also play a role in their sequestration, thereby inhibiting/attenuating their possible deleterious effects^54^. This track has not been exploited yet to explain the level of AMP sensitivity of deep-rough mutants. As an example, we identify an *overabundance* of OppA in the outer membrane of JW3596 (25 times more than in the WT) and JW3606 (18 times more than the WT) as revealed by our proteomics analysis (Fig. 8). This protein is an essential oligopeptide binding protein involved in the uptake of peptides (2 to 18 amino acids depending on the organism) from the environment before transportation to the cytoplasm^55^. The possible implication of OppA in the internalisation of low-molecular AMPs has only been recently highlighted^56^. Maio *et al*. showed that the tetrapeptide GE81112 produced by a *Streptomyces* sp. is transported by the oligopeptide permease system (Opp) of *E. coli* and *B. subtilis*^56^. As a consequence, this AMP, particularly effective on Gram-positive and Gram-negative bacteria in minimal broth media, becomes essentially inactive in rich-peptide media due to competition for transport into the cells.

Figure 8 evidences that most of the well known receptor-dependent AMPs like bacteriocins produced by- and lethal to *E. coli* (*i.e*. colicin and microcin), are *less abundant* on the outer membrane of the deep-rough mutants compared to WT cells (Fig. 8). This is the case for BtuB - receptor of colicin A and nine colicins E^57^ - in JW3596 and JW3601, and for OmpW - receptor of colicin S4^58^ -, FepA - receptor of colicin B and D^59,60^, and FhuA - receptor of colicin M^61^ and microcin J25^62^- in JW3596, JW3601 and JW3606. Unfortunately, the genome-wide screening of colicin activity against mutants from the Keio collection previously performed by Sharma *et al*. did not allow to technically reveal the colicin tolerance of deep-rough mutants, probably because of the high colicin concentrations used in their screening procedure^63^.

#### Susceptibility to hydrophobic antibiotics

Another feature of deep-rough mutants is their hypersensitivity to novobiocin and other hydrophobic antibiotics such as actinomycin D, erythromycin and rifampicin^4,27,64^. Recent screening of the entire *E. coli* Keio collection in terms of response to 22 antibiotics revealed an increased sensitivity of JW3596 (Δ*rfaC*) and JW3606 (Δ*rfaG*) to 14 antibiotics and to 9 antibiotics, respectively, compared to WT reference cells^17^ whereas the strain JW3601 (Δ*rfaJ*) was not revealed by the screening procedure, which suggests different sensitivity patterns to antibiotics between mutants with inner-core and outer-core truncated LPS. Along the same lines, Chang *et al*. compared novobiocin susceptibility of the Keio strains JW3596 and JW3606 with that of the WT cells, and reported a 4-fold increase in novobiocin sensitivity for JW3596 compared to JW3606^7^. As argued by several authors, sensitivity of deep-rough mutants should be connected to changes in bacterial cell surface hydrophobicity and permeability^3^. The generally observed hypersensitivity of JW3596 and JW3606 to hydrophobic compounds is again likely favored by their significant membrane destabilization evidenced by electrokinetics (Fig. 2) and AFM analyses of their nanomechanical features(Figs 5 and 6).

As a conclusion of that section, it is found that strains with inner-core alteration of their LPS (*i.e*. JW3606 and JW3596) exhibit enhanced sensitivity to hydrophobic antibiotics (and, similarly, to AMP, see previous section), which correlates with their enhanced membrane destabilization highlighted in the current study.

The peculiar properties of deep-rough mutants, which encompasses a destabilization of their protective envelope barrier with increasing the extent of LPS truncation, also provide some hints to apprehend their hypersensitivity to several *hydrophilic antibiotics*^4,17^, to *anionic and cationic detergents*^3–5^, *bile salts*^63^, *dyes*^3–5^, *fatty acids*^4,65^, to *phenols* and *polycyclic hydrocarbons*^4,32,65^. In addition, the higher cell turgor pressures measured for the mutants with inner core altered LPS, qualitatively correlate with their *low transformation efficiencies* compared to that of the WT reference^7,19^, a difference that possibly originates from limitation of DNA intracellular entry and/or promotion of cell lysis during heat or electric shock. Analysis of membrane proteins in the three *rfa*-mutants (Fig. S1) reveals some other phenotypic characteristics of these strains, such as a modification of their chemotaxis toward amino acids due to change in the abundance of Tar and Tsr receptors. The overabundance of SecA (5.15 to 7.91 more abundant in *rfa*-mutants compared to WT), which is an ATPase required for the translocation of proteins into vesicles of *E. coli*^66,67^, also suggests an effect of *rfa*-mutations on cell vesiculation process. This element is further supported by results recently obtained by Kulp *et al*. showing that mutations leading to truncated LPS induce cell hypervesiculation^68^. The overabundance of ElaB (21.58 to 32.06 more abundant in *rfa*-mutants compared to WT) should be further emphasized as it reflects a deep stress in these mutants.

## Conclusions

In the current study, a suite of complementary experimental techniques (*i.e*. microelectrophoresis, Atomic Force Microscopy (AFM) and Isobaric Tag for Relative and Absolute Quantitation (iTRAQ) of proteins) were used for probing cell envelope properties of several *rfa*-gene mutants displaying peculiar deep-rough phenotypes. Compared to the WT reference, these mutants exhibit significant physical and biochemical modifications of their surface, which includes changes of their surface roughness, envelope elasticity and permeability, internal turgor pressure and proteic composition. This finding is especially observed for mutants with altered LPS in the inner core probably due to the loss of core phosphorylation essential for outer membrane stability. It is further shown that the abundance of several outer membrane proteins involved in the adhesion/transportation of bacteriophages and several antibacterial substances significantly depends on the LPS truncation degree. Altogether, these results provide an original route for examining and discussing the respective reactivities of deep-rough phenotype with respect to *e.g*. antimicrobial agents, may they be bacteriophages, cationic antimicrobial peptides or antibiotics.

## Methods

### Strains and culture conditions

*Escherichia coli* strains BW25113 (hereafter referenced as wild type WT) and the knock-out mutants JW3601 (Δ*rfaJ*), JW3606 (Δ*rfaG*) and JW3596 (Δ*rfaC*) were obtained from the Coli Genetic Stock Center, Yale University. Position of the mutations in the *rfa* operons and structure of the LPS resulting from these mutations^12^ are reported in Fig. 1. Knock-out mutations were checked by PCR before freezing at −80 °C in 50% glycerol solution. For experiments, strains were streaked from glycerol stocks on LB agar plates. Overnight precultures were realized by inoculating single colonies in 4 mL of M9 minimal medium (per liter: MgSO_4_ 1 mM, CaCl_2_ 0.1 mM, Thiamine 10 µg/mL, 2 g/L Glucose, Proline 20 µg/mL, Uridine 25 µg/mL) and incubating at 37 °C under 150 rpm agitation. The next day, 1 mL precultures volume was introduced in 100 mL of M9 minimal medium and incubated at 37 °C and 150 rpm until DO_600nm_ reached 0.4–0.6. Then, cells were washed twice by centrifugation-resuspension (5000 × g for 8 min) in sterile 10 mM KNO_3_ and adjusted to OD_600nm_ 0.4. Note that for cultures of WT cells on LB or in M9 media, no antibiotics were added, whereas for knock-out mutants, media were always supplemented with 30 mg/L of Kanamycin.

### Electrokinetics

Cell suspensions prepared as detailed above were diluted at an OD_600nm_ of 0.04 in KNO_3_ electrolyte solutions with concentration ranging from 1 mM to 500 mM. The electrophoretic mobility of the bacterial cells was then measured as a function of electrolyte concentration at natural pH ∼6 and room temperature using a Zetaphoremeter IV (CAD Instrumentations, Les Essarts le Roi, France). Electrophoretic mobility evaluation consisted in following the displacements of bacteria in a quartz Suprasil® rectangular capillary when subjected to a constant direct-current electric field (around 800 V/m) *via* tracking the reflection by the bacteria of a laser beam at a 90° angle by means of a Charge-Coupled Device camera^69^. Trajectories were recorded in real time and were processed with an image analysis software to derive (Gaussian-like) mobility distribution from which mean value was extracted. For each ionic strength condition tested, cell migration was recorded on three aliquots of the same bacterial suspension and two independent assays were further performed according to the aforementioned procedure. Each mobility data point obtained thus corresponds to the average of six electrokinetic measurements.

### Atomic force microscopy measurements

Bacteria from the cell suspension prepared as detailed above were first deposited on a cleaned borosilicate glass slide previously covered with a layer of polyethyleneimine (PEI) (Sigma, Mw = 750 000 g/mol) cationic polymer upon immersion in 0.2% PEI solution for 30 min. After a few minutes, the glass slide was rinsed with 1 mM KNO_3_ solution to remove unbound or loosely bond bacteria from the surface. The living bacteria fixed on the surface were then maintained in a 1 mM KNO_3_ environment during AFM experiments performed at ambient temperature and natural pH. Measurements were conducted with a FastScan Dimension Icon with Nanoscope V controller (Bruker) operating in Peak Force tapping mode in fluid medium. This mode has the advantage to provide simultaneously both cell surface images with *ca*. 20 nm resolution (size of the AFM tip) and loading force measurements as a function of the AFM tip-to-cell surface separation distance (Fig. 4), the latter measurements being then converted into force *versus* indentation depth curves. The AFM probes used were NPG Silicon Nitride tips (with a nominal value of the spring constant of 0.32 N/m reported by the manufacturer). Prior to each measurement, a calibration was performed on a rigid substratum in order to determine the deflection sensitivity (nm/V) of the AFM tip. In turn, this leads to the accurate evaluation of the cantilever spring constant following thermal noise method^70^, with values in the range 0.40 ± 0.2 N/m. Force measurements were recorded during both the ‘approach’ and ‘retraction’ of the AFM tip to/from the bacterial surface. The pixel-by-pixel approach/retraction curves were performed at the center of the cell with 500 nm scan size (corresponding to 256 × 256 local force measurements on the investigated cell surface zone) at 1 Hz scan rate and tip velocity displacement of 1 µm/s. Reproducibility/statistics of the nanomechanical data was assessed from force measurements conducted on *ca*. 15 different bacteria per strain, in detail 2 to 3 different bacteria per cell culture and 5 to 6 different cell cultures. The Young modulus *E* of the cell envelope and the internal Turgor pressure (which is related to the cell spring constant also termed cell stiffness and denoted as *k*_cell_^24^) for the strains of interest were derived from AFM force curves following the procedure illustrated in Fig. 4 and inspired from previous reports^71^. Briefly, the Hertz model corrected by Dimitriadis *et al*.^72^ was selected here to express the force as a function of indentation depth, while the slope of the linear compliance regime of the AFM force curves is determined by *k*_cell_ (Fig. 4). Spatial mapping of these two defining cell properties was realized from force measurements performed on a 256 by 256 pixels (which corresponds to a total of 65536 force curves) scanned surface area *via* application of a 5 nN loading force. Cell surface roughness (Fig. 3B) was evaluated from 49 to 52 cell images for each strain. In detail, the measurements were conducted on 3 to 12 different bacteria (probed cell surface area 500 × 500 nm^2^) per cell culture, and statistics was established from measurements on 7 to 11 different cell cultures. The time required to evaluate the surface roughness from a recorded image collected for a single cell is *ca*. 30 seconds. That necessary for derivation of the spatially-resolved Young modulus and cell stiffness (treatment of 65536 force curves on 500 × 500 nm^2^ cell surface area according to Hertz-Dimitriadis contact mechanics formalism, Fig. 4) is *ca*. 25 minutes. This comparison explains why our total number of measurements adopted for the establishment of the nanomechanical data is somewhat lower than that for surface roughness data. It is stressed however that the statistics for each of these evaluations (cell surface roughness and nanomechanical properties) conform well to- and is even larger than- that reported in many AFM reports on biological particles^28^. The relative evolution of cell surface roughness from one strain to another (Fig. 3B) being relatively weak compared to that for the mechanical parameters (there is a *ca*. 2-fold evolution of the Young modulus and *k*_cell_ over the range of cell strains examined, Fig. 6), the statistics of surface roughness data was increased to get a solid picture of the trends.

### Cell membrane protein extraction

Membrane proteins were isolated using sodium carbonate extraction^29,30^. Briefly, 10^12^ cells (counted by flow cytometry) in log phase of growth (DO_600nm_ approximately 0.5) were pelleted and resuspended in 6 mL of 50 mM Tris/HCl at pH 7.3 containing 0.7 mg of DNase I. Cells were broken in a French press twice at 1 kBar and centrifuged at 2500 × *g* for 10 min. The supernatant containing broken cells was diluted 10 times with ice cold 0.1 M sodium carbonate (pH 11) and stirred slowly at 4 °C for 1 h. Carbonate-treated membrane were pelleted by ultracentrifugation at 115000 × *g* for 1 h in a Kontron TFT 50.38 rotor, washed in 50 mM Tris/HCl pH 7.3, and the final pellet was collected by ultracentrifugation at 115000 × *g* for 20 min. Cell membranes were dissolved in 300 μL of 500 mM TEAB (Tetra Ethyl Ammonium Bicarbonate)/0.1% SDS and denatured 10 min at 95 °C. Protein concentration was estimated by the Bradford method with bovine gamma globulin as standard (BioRad) and protein quality was checked by protein separation on a SDS-PAGE 12% and by staining with Coomassie blue R250^73^. Protein concentration was adjusted to 3 μg/μL with 500 mM TEAB/0.1% SDS/20% Acetonitrile (ACN).

### iTRAQ labeling, peptide fractionation and LC-MS/MS analysis

#### Isobaric peptide labeling

A volume corresponding to 90 μg of proteins for each sample was reduced with dithiotreitol from Sigma (30 mM DTT, 30 min at 56 °C), alkylated (60 mM Chloroacetamide from Sigma, 30 min at 25 °C), and digested (1 μg sequencing grade trypsine from Promega, overnight at 30 °C). The pH was checked to ensure complete digestion. Samples were subsequently dried in a vacuum concentrator (Eppendorf), resuspended in 35 μl water and labeled with iTRAQ reagents according to the protocol of the kit (Applied Biosystems). Samples were labeled with distinct isobaric tags. In detail, WTA, WTB, 3596A, 3596B, 3601A, 3601B, 3606A and 3606B samples were labeled with 113, 114, 115, 116, 117, 118, 119 and 121, respectively. Labeled samples were then combined.

#### IEF off-gel fractionation

The iTRAQ reagent excess and detergent were eliminated by using an SCX column (Strong Cation exchange, ABSciex). Briefly, SCX column was prewashed with 2 mL of cleaning buffer (25% ACN, 10 mM KH_2_PO_4_, 1 M KCl pH 3.0) and equilibrated with 2 mL of loading buffer (25% ACN, 10 mM KH_2_PO_4_, pH 3.0). Sample was resuspended using 1 mL of loading buffer acidified with 20 μl of KH_2_PO_4_ 10% to obtain a solution of pH 3.0, further percolated on column, and then washed with 2 mL of loading buffer. Retained peptides were eluted with 500 μl of elution buffer (25% ACN, 10 mM KH_2_PO_4_, 350 mM KCl, pH 3.0). Eluted peptides were desalted by using a Sep-Pak C18 column (Waters). Briefly, C18 column was activated with 3 mL of 90% ACN, 0.1% TFA (Trifluoroacetic acid; Fluka) and equilibrated with 3 mL of 0.1% TFA. Peptides were percolated on column, retained fraction was washed with 2 mL of 0.1% TFA, eluted with 1 mL of 70% ACN, 0.1% TFA and then dried in a vacuum concentrator. Peptides were prepared for an Off-gel isoelectrofocalisation on 13 cm pH 3 to 10 strip as indicated in Agilent 3100 Off-Gel fractionator kit-quick start guide. After focusing, each fraction was collected. To extract peptides trapped in strip gel, 200 μL of 50% methanol 1% formic acid were added to each tank of the frame and incubated for 30 min. Methanol-extracted peptides were pooled with their respective fraction, then dried in a vacuum concentrator and finally resuspended in 100 μL of 10% ACN and 0.1% TFA solution.

#### LC-MS/MS

Analyses were performed on 1 μg of each fraction using an Ultimate 3000 Rapid Separation liquid chromatographic system, coupled with a hybrid Linear Trap Quadrupole (LTQ) Orbitrap Velos mass spectrometer (Thermo Electron). Briefly, peptides were loaded and washed on a C18 reverse phase precolumn (3 μm particle size, 100 A pore size, 75 μm i.d., 2 cm length from Thermo Fisher Scientific). The loading buffer contains 98% H_2_O, 2% ACN and 0.1% TFA. Peptides were then separated on a C18 reverse phase resin (2 μm particle size,100 A pore size, 75 μm i.d.,15 cm length) with a 97 min ’effective‘ gradient from 100% A (0.1% formic acid and 100% H_2_O) to 10% B (80% ACN, 0.085% formic acid and 20% H_2_O) in 33 min then to 40% B in 64 min. The mass spectrometer acquired data throughout the elution process and operated in a data dependent scheme with full MS scans acquired with the orbitrap, followed by up to 10 LTQ MS/MS CID spectra and 10 LTQ MS/MS HCD on the most abundant ions detected in the MS scan. Acquisition Trigger settings were: full MS (AGC: 1.10^6^, resolution: 6.10^4^, m/z range 400–2000, maximum ion injection time (MIIT): 500 ms); MS/MS (minimum signal threshold: 500, isolation width: 2 Da, dynamic exclusion time setting: 60 s, Ion Trap MSn AGC Target: 5.10^3^ and MIIT: 200 ms, FTMS MS^n^ AGC Target: 5.10^4^ and MIIT: 200 ms). The fragmentation was permitted for precursor with a charge state of 2 and 3.

#### Spectral processing and database searching

The software used to generate.mgf files was Proteome discoverer 1.3. The threshold of Signal to Noise for extraction values was 3. The node Spectrum grouper was used to group HCD and CID scans. The.mgf files were submitted to Protein Pilot version 4. The adopted database was *Escherichia coli* K12 extract from database Uniprot. FDR calculations were performed using a reverse database. Search effort was set to Rapid ID and instrument used was Orbi-FT MS (1–3 ppm), LTQ MSMS. Analysis was performed using Trypsin as enzyme and carbamidomethylation as fixed modification. For iTRAQ quantification, bias correction and background correction were applied to Protein pilot research. A protein was considered to be significantly identified when 2 or higher confidence (>95%) unique peptides were assigned, the protein identification had to have a 5% local False Discovery Rate. The relative quantification was performed using the Wild Type biological duplicates as reference samples. Ratios of fold change and the associated p-value were calculated for each biological duplicate (mutant/WT), then the median value of the four ratios was obtained. Proteins with ratio fold changes ≥2 or ≤0.5 and 3 p-values ≤ 0.05 were considered to be significantly differentially expressed. Functional annotations of the proteins were conducted using the Blast2GO program against the non-redundant protein database (NR; NCBI). Gene/protein ontology analyses were carried out using the functional annotation tool from the Database for Annotation, Visualization and Integrated Discovery (DAVID 6.7) available online through the National Institute of Allergy and Infectious diseases (NIAID)^74–76^. The EASE score, a modified Fisher exact *p*-value, adopted to measure the protein enrichment in annotation term was set below 0.01.

Western blot analysis and experimental procedure used for validation of the iTRAQ results are presented in Supplementary Information (Section SI-3, Fig. S3).

## Supplementary information

Supplementary Information

Table S1
