# Supplementary material for: Pleiotropic effects of rfa-gene mutations on Escherichia coli envelope properties
Source: Sci Rep. 2019 Jul 4;9:9696. doi: 10.1038/s41598-019-46100-3 (PMC6609704; doi:10.1038/s41598-019-46100-3)
Supplement: Supplementary file 1 — Supplementary Information [file 41598_2019_46100_MOESM1_ESM.pdf]

SUPPLEMENTARY INFORMATION

Pleiotropic effects of *rfa*-gene mutations on *Escherichia coli* envelope properties

Christophe Pagnout,*[,1] Bénédicte Sohm,[1] Angélina Razafitianamaharavo,[2] Céline Caillet,[2] Marc Offroy,[2] Marjorie Leduc,[3] Héloïse Gendre,[2] Stéphane Jomini,[4] Audrey Beaussart,[2] Pascale Bauda,[1] Jérôme F. L. Duval[2]

[1] Université de Lorraine, LIEC, UMR7360, Campus Bridoux, Metz F-57070, France.

[2] Université de Lorraine, LIEC, UMR7360, Vandoeuvre-lès-Nancy F-54000, France.

[3] Plateforme protéomique 3P5, Inserm U1016-Institut Cochin, Université Paris Descartes, MICUSPC, Paris, France.

[4] ANSES, 94701 Maisons-Alfort Cedex, France

*Corresponding author: christophe.pagnout@univ-lorraine.fr

SI-1. Relative abundance of 17 selected proteins in JW3601, JW3606 and JW3596.

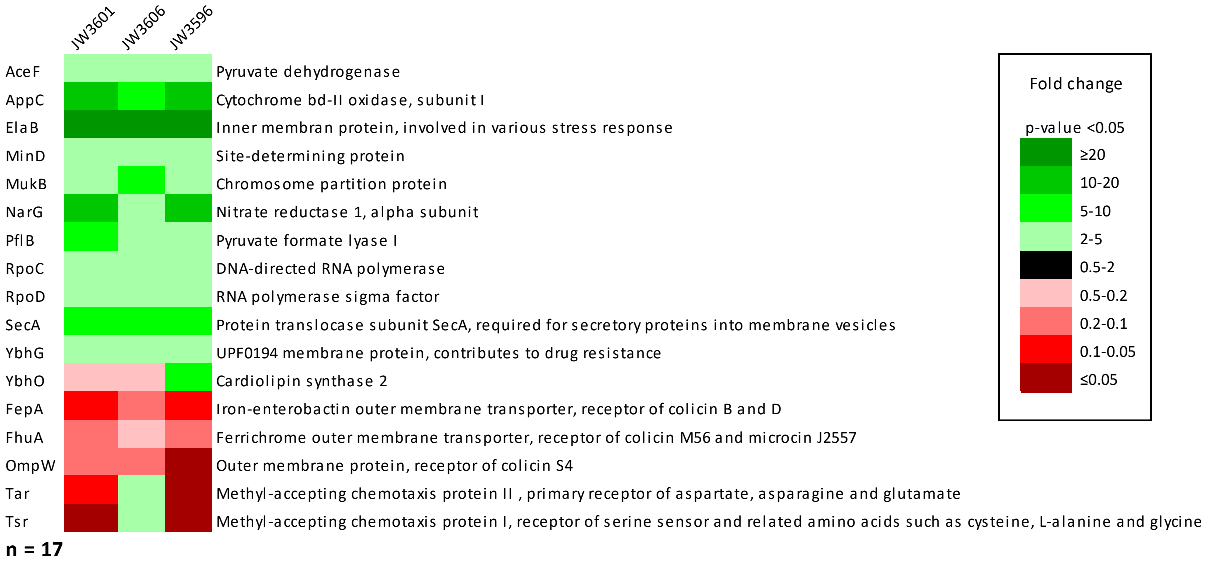

Figure S1. Heat map view of 17 selected proteins found to be more or less abundant in JW3601, JW3606 and JW3596 as compared to the WT reference (BW25113). The increased and decreased abundance of these proteins are indicated by the range of green and red intensities, respectively.

SI-2. Detailed iTRAQ results (Table S1: attached .xls file)

Table S1 reports N-identified proteins, ordered in decreasing scores, identified from the iTRAQ results obtained on the membrane proteins extracted from the strains JW3596, JW3601, JW3606 and from the WT reference (BW25113). The table includes the list of iTRAQ ratios, protein scores, percentages of sequence coverage, gene accession symbol, description and numbers of identified peptides for each protein.

SI-3. Western blot analysis for iTRAQ data validation.

Cell wall proteins were extracted as described in the main text. Cytoplasmic proteins were extracted as follows: cells were broken in a French press twice at 1 kBar and centrifuged at 2500 × *g* for 10 min. Then, supernatant was centrifuged at 12000 × *g* for 20 min and diluted v/v in NP40 buffer (50 mM Tris/HCl pH 7.3, 150 mM NaCl, 1 mM EDTA, 0.1% SDS and 1% NP40). Both cell wall and cytoplasmic proteins (10 µg) were separated by SDS-PAGE, transferred to a nitrocellulose membrane and stained with Ponceau S 0.1 % (w/v), and acetic acid 5% (v/v) for verification. Membranes were probed with rabbit polyclonal antibodies specific for Flagellin (Abcam, dilution 1/15000) or for Methyl-accepting chemotaxis protein II TAR (antibodies-online GmbH, dilution1/10000) for confirmation of ITRAQ results. Membranes were also probed with mouse antibody specific for *E.coli* DnaK (clone 8E2/2, dilution 1/500). DnaK is a cytoplasmic protein detected only in cytoplasmic extract, which therefore helped in confirming the purity of the membrane extract. Secondary antibodies were horseradish peroxidase coupled anti-rabbit IgG (dilution 1/10000) or anti-mouse (dilution 1/2000) IgG and the signal was detected upon chemoluminescence measurement (BioRad) and use of Imager 600 (Amersham).

Figure S2 shows western blot analysis for both membrane and cytoplasmic protein extracts of strains JW3606, JW3601, JW3596 and the WT hybridized with anti-FliC, anti-Tar and anti-DnaK antibodies. Results obtained are consistent with the iTRAQ data and confirm the highest abundance of proteins FliC and Tar in JW3606 compared to JW3601, JW3596 and the WT cells. The only presence of DnaK (a cytoplasmic protein) in the cytoplasmic extracts confirm the purity of the membrane extracts.

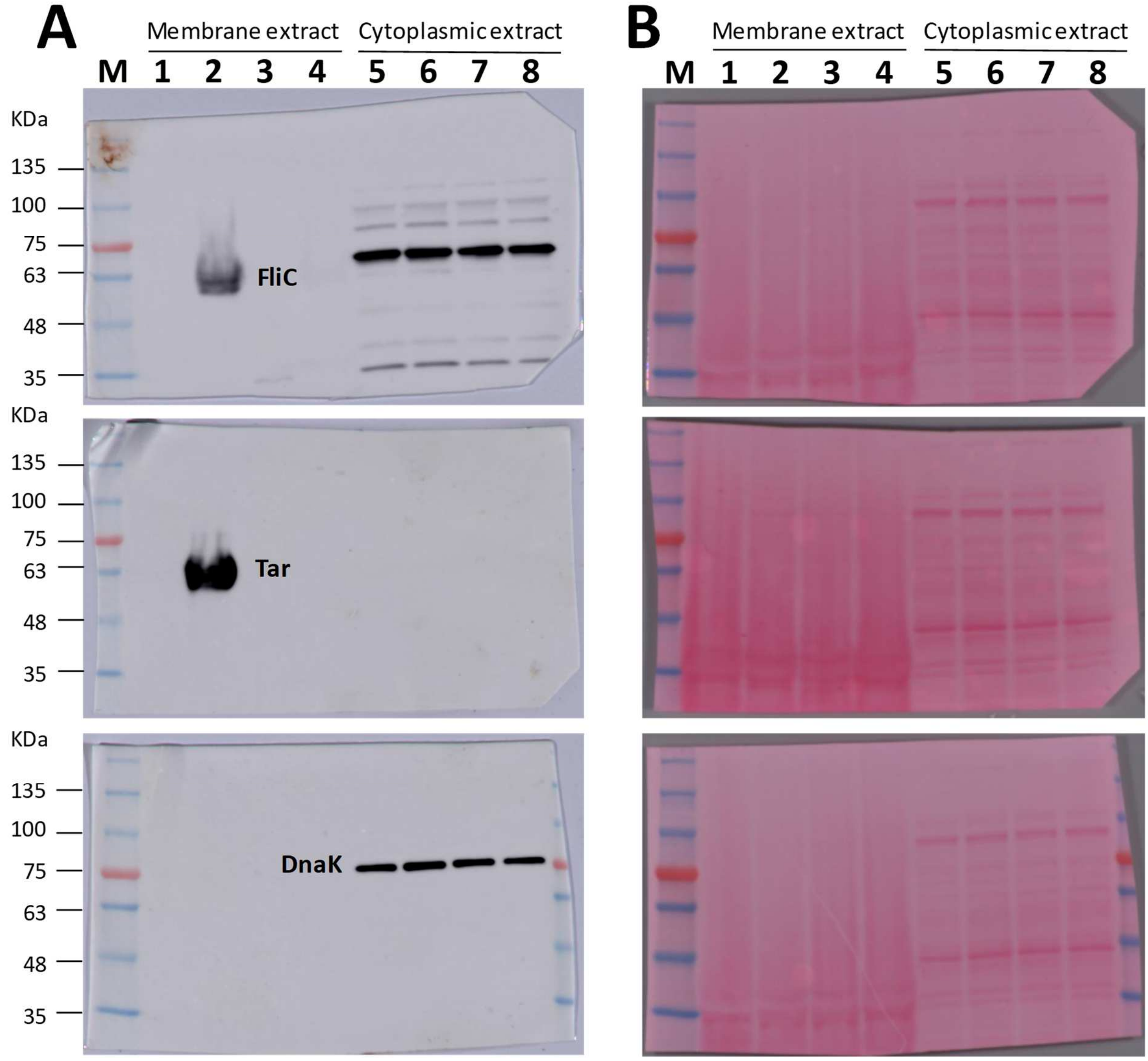

Figure S2. Western blot analysis for validation of iTRAQ results (A) Cell membrane extracts (10 µg) in TEAB/SDS buffer previously used for iTRAQ experiments (lines 1 to 4) and cytoplasmic extracts in NP40 buffer (lines 5 to 8) probed with three different antibodies, namely anti-FliC, anti-Tar and anti-DnaK. (B) Ponceau staining of the membranes used for immunoblotting. Lines 1 and 5, BW25113 (WT); Lines 2 and 6, JW3606; Lines 3 and 7, JW3601; Lines 4 and 8, JW3596; M, molecular mass ladder (in KDa).

SI-4. Outer membrane protein extraction.

Specific outer membrane extraction was performed as described previously by Yethon et *al.* (2000). A given amount of cells (fixed upon measurement of the optical density at 600 nm) was harvested by centrifugation of 100 ml exponential growth cultures (OD_{600nm}= 0.6), resuspended in 20 mM Tris buffer (pH 8), and lysed by passage through a French pressure cell. After removal of cell debris (5000 × *g* for 10 min), the total membrane fraction was collected by centrifugation (100.000 × *g* for 2 h) and then resuspended in 2% Sarkosyl. The Sarkosyl-insoluble outer membrane fraction was collected by centrifugation, washed a second time in 2% Sarkosyl, and centrifuged again. The

resulting pellet was resuspended in 1 ml of 20 mM Tris buffer (pH 8), denatured in Laemmli buffer, analyzed on a SDS-PAGE 12% and Coomassie stained.

Indicated bands (**Figure S3**) were manually excised from gels and were cut into cubes. Tryptic digestion and mass spectrometry analysis were performed by the proteomic platform 3P5 (Université Paris Descartes, Institut Cochin, Paris). In-gel digestion was carried out with trypsin: samples were destained twice with a mixture of 100 mM ammonium bicarbonate (ABC) and 50% (vol/vol) acetonitrile (ACN) for 20 min at room temperature and then dehydrated using 100% ACN for 20 min, before being reduced with 25 mM ABC containing 10 mM DTT for 1 h at 56 °C and alkylated with 55 mM iodoacetamide in 25 mM ABC for 30 min in the dark at room temperature. Gel pieces were washed twice with 25 mM ABC and dehydrated (twice, 20 min) with 100% ACN. Gel cubes were incubated with sequencing grade-modified trypsin (Promega, USA; 12.5 ng/µl in 40 mM ABC with 10% ACN, pH 8.0) overnight at 37 °C. After digestion, peptides were extracted twice from gel pieces with a mixture of 50% ACN – 5% formic acid (FA) and then with 100% ACN. Extracts were dried using a vacuum centrifuge concentrator plus (Eppendorf).

Mass spectrometry (MS) analyses were performed on a Dionex U3000 RSLC nano-LC system coupled to a Q-Exactive Plus mass spectrometer (Thermo Fisher Scientific). After drying, peptides were solubilized in 7 µL of 0.1 % trifluoroacetic acid (TFA) containing 10 % acetonitrile (ACN). One µL was loaded, concentrated and washed for 3 min on a C_{18} reverse phase precolumn (3 µm particle size, 100 Å pore size, 75 µm inner diameter, 2 cm length, Thermo Fisher Scientific). Peptides were separated on a C_{18} reverse phase resin (2 µm particle size, 100 Å pore size, 75 µm inner diameter, 25 cm length, Thermo Fisher Scientific) with a 20 minutes gradient starting from 99 % of solvent A containing 0.1 % FA in H_2O and ending in 40 % of solvent B containing 80 % ACN, 0.085 % FA in H_2O. The mass spectrometer acquired data throughout the elution process and operated in a data-dependent scheme with full MS scans acquired with the Orbitrap, followed by up to 10 MS/MS HCD fragmentations in the Q-Exactive Plus on the most abundant ions detected. Resolution was set to 70,000 for full scans at AGC target 3.0e6 within 100 ms maximum injection ion time (MIIT). The MS scans spanned from 400 to 2000 m/z. Precursor selection window was set at 4 m/z, and MS/MS scan resolution was set at 17,500 with AGC target 1.0e5 within 100 ms MIIT. HCD Collision Energy was set at 30 %. Dynamic exclusion was set to 15 s duration. For the spectral processing, the software used to generate .mgf files was Proteome Discoverer 1.4 (ThermoFisher Scientific). The mass spectrometry data were analyzed using Mascot v2.5 (Matrix science) on *Escherichia coli* (23,076 sequences) from the SwissProt databank containing 558,590 sequences; 200,544,181 residues (October 2018). The enzyme specificity was Trypsin's and up to 1 missed cleavage was tolerated. The precursor mass tolerance was set to 4 ppm and the fragment mass tolerance to 20 mmu for Q-Exactive Plus data.

Carbamidomethylation of cysteins was set as constant modification and oxidation of methionines was set as variable modification.

Figure S3 shows the SDS-PAGE analysis of the outer membrane fractions of BW25113 (WT), JW3596, JW3601 and JW3606 (in that order from left to right) and reveals that the dramatic decrease (more than 90%) of porins OmpA, OmpF and OmpC argued by several authors (Nikaido, 1979; Nikaido and Vaara, 1985; Parker et al., 1992; Schnaitman and Klena, 1993) is not supported by our experimental data. OMP extractions and SDS-PAGE experiments were performed in triplicates and no significant difference was observed between OmpA, OmpF and OmpC (only one representative SDS-PAGE gel is presented in **Fig. S3**).

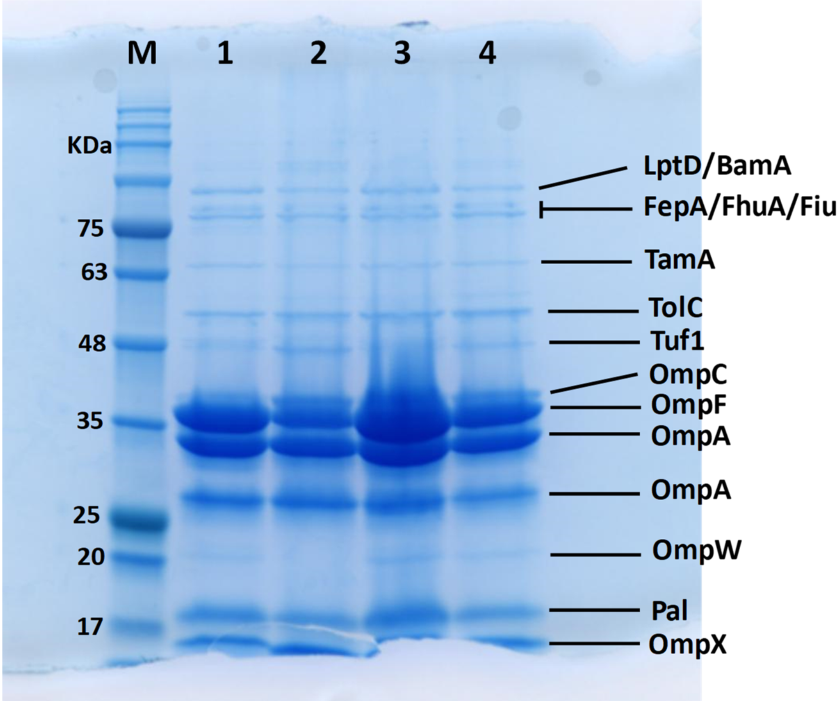

Figure S3. Coomassie brilliant blue-stained SDS-PAGE gel (12%) of outer membrane fractions. M, Molecular mass ladder (KDa); 1, BW25113 (WT); 2, JW3596; 3, JW3601; 4, JW3606. The main outer proteins that are unambiguously identified by mass spectrometry are indicated on the right as the top protein hit identified in the gel piece.

SI-5. Strain complementation experiments.

Complementation experiments were performed using the ASKA plasmids provided by the National BioResource Project (NIG, Japan): *E. coli.* Plasmids pCA24N::*rfaJ*, pCA24N::*rfaG*, pCA24N::*rfaC* were extracted from the ASKA clone(-) JW3601, JW3606 and JW3596 using the Macherey-Nagel NucleoSpin Plasmid kit (Fisher Scientific, France) and transformed to the respective Keio stains JW3601, JW3606 and JW3596. As a control, the empty plasmid pCA24N was transformed in the WT strain BW25113. Thermo-competent cells were prepared and transformed according to standard procedures (Green and Sambrook, 2012) and spread on LB plates containing 25 µg/ml

chloramphenicol and 30 µg/ml Kanamycin (only chloramphenicol for BW25113 pCA24N). Clones were selected, verified by PCR, and cultured for further analyses in M9 broth supplemented with 1.0 mM isopropyl β-d-1-thiogalactopyranoside (IPTG) and the appropriate antibiotics.

Table S2 presents the Minimum Inhibitory Concentrations (MICs) of sodium dodecyl sufate (SDS) obtained for *rfa*-mutants and their complemented derivatives, namely JW3601, JW3606 and JW3596 carrying the plasmids pCA24N::*rfaJ*, pCA24N::*rfaG*, pCA24N::*rfaC*, respectively. MIC of SDS was also obtained for the reference strain BW25113 (WT) carrying or not the empty control plasmid pCA24N. These values correspond to the lowest concentrations of SDS that inhibit the growth of strains. The cell growth conditions adopted for BW25113 and *rfa*-mutants are as specified in the *strains and culture conditions* section of the main text (M9 medium growth, kanamycin for mutants). For complemented strains, growth conditions are as specified above (M9 medium growth, chloramphenicol, kanamycin for mutants, and IPTG). Results obtained reveal that complementation totally suppresses the SDS sensitivity of JW3606 and JW3596, which is a peculiar feature of inner core truncated LPS-mutants. These data are in good agreement with the expected results and confirm that for these two strains complementation restores the wild-type phenotype.

Table S2. Minimum Inhibitory Concentrations (MICs) of SDS.

	MIC of SDS (mg/mL)
BW25113 (WT)	10-100
JW3601	10-100
JW3606	0.1-1
JW3596	0-0.1
BW25113 pCA24N	10-100
JW3601 pCA24N::*rfaJ*	10-100
JW3606 pCA24N::*rfaG*	10-100
JW3596 pCA24N::*rfaC*	10-100

Following the methodology detailed in the main text, **Figure S4A** displays surface roughness (R_{surface}) of cells of the complemented strains (displaying a morphology similar to that given in **Fig. 3A** of the main text) denoted hereafter as JW3601*, JW3606* and JW3596* carrying the plasmids pCA24N::*rfaJ*, pCA24N::*rfaG*, pCA24N::*rfaC*, respectively, and of the reference strain BW25113* (WT*) **transformed with the empty plasmid pCA24N**. The cell growth conditions adopted for each strain is specified above (*i.e.* M9 growth medium, presence of chloramphenicol, of kanamycin for mutants and IPTG). Adopting the procedure detailed in the main text (**Fig. 4**), Young modulus (E) and cell spring constant (k_{cell}) were further evaluated and results are reported in **Figures S4B** and **S4C**, respectively.

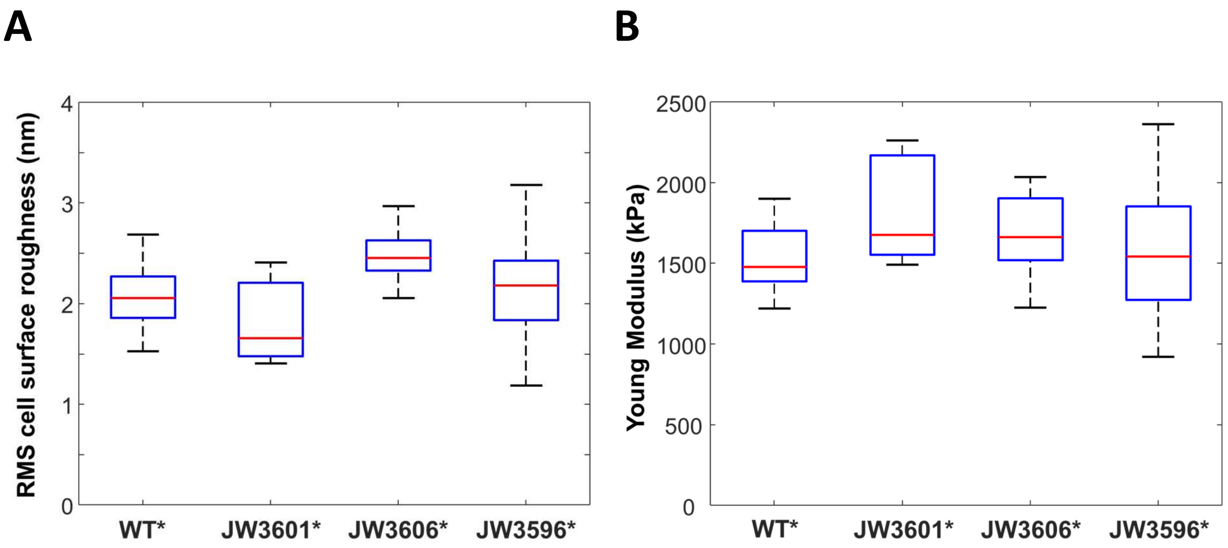

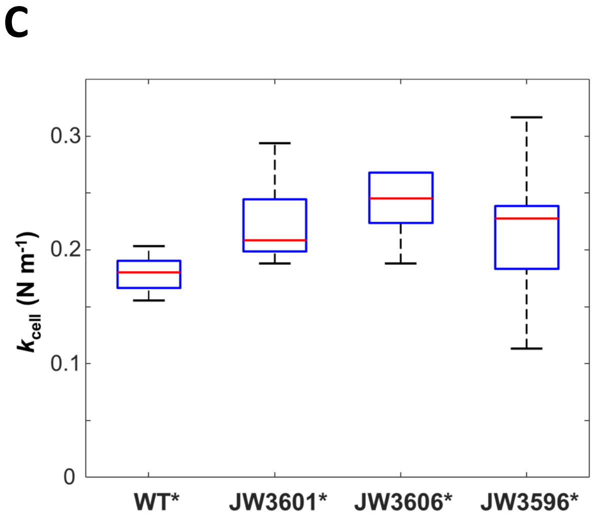

Figure S4. Dependence of the RMS cell surface roughness (A), Young modulus (B) and cell spring constant (C) pertaining to the complemented strains WT* (BW25113 pCA24N), JW3601* (JW3601 pCA24N::*rfaJ*), JW3606* (JW3606 pCA24N::*rfaG*) and JW3596* (JW3596 pCA24N::*rfaC*). Data derive from measurements conducted on 2 to 8 cells per cell culture and 2 to 4 different cell cultures. Overall, 10 to 15 cells per strain were considered for evaluation of $R_{surface}$, E and k_{cell}.

The number of cells examined for evaluation of cells surface roughness and nanomechanical properties is here 10 to 15, in detail 2 to 8 cells per cell culture and 2 to 4 different cell cultures were considered. Altogether, the AFM-derived results show that the surface roughness and the nanomechanical features of JW3601*, JW3606*, JW3596* and WT* are comparable, and that they significantly differ from those discussed in the main text for the WT and deep-rough mutants cells pictured in **Fig. 3A**. It is not unexpected that the similar cell surface properties of JW3601*, JW3606*, JW3596* and WT* are different from those of the BW25113 control strain **lacking the empty plasmid pCA24N**, recalling that the different growth conditions adopted with and without pCA24N (presence/absence of chloramphenicol and IPTG) impact on *e.g.* the rate of cells division, the ductility of their envelop, and their surface elasticity. The key result here is that the surface characteristics of JW3601*, JW3606*, JW3596* and WT* are of the same magnitude and that there is no clear sign of dependence on cell strain. In particular, the values of $R_{surface}$ obtained for JW3601*, JW3606*,

JW3596* and BW25113* are all within a range that is significantly narrower than that of the data collected for JW3601, JW3606, JW3596 and BW25113 (**Fig. 3B**) with no clear evidence of a marked dependence on cell strain. In addition, whereas a *ca*. two-fold increase in E and k_{cell} was measured over the range of non-complemented strains (**Fig. 6**), the complemented strains JW3601*, JW3606*, JW3596* and BW25113* are defined by a nearly constant Young modulus (1.5-1.7 MPa) and by a k_{cell} comprised in the restricted range 0.18-0.24 N m^{-1} (median values).

References:

Green, M. R., Sambrook, J. Molecular cloning: a laboratory manual, 4th edn. Cold Spring Harbor Press, New York (2012).

Nikaido, H. Permeability of the outer membrane of bacteria. *Ang Chemie* **18**, 337–420 (1979).

Nikaido, H. & Vaara, M. Molecular basis of bacterial outer membrane permeability. *Microbiol. Rev.* **49**, 1–32 (1985).

Parker, C. T. *et al.* Role of the *rfaG* and *rfaP* genes in determination of the lipopolysaccharide structure and cell surface properties of *Escherichia coli* K-12. *J Bacteriol* **174**, 2525–2538 (1992).

Schnaitman, C. A. & Klena, J. D. Genetics of lipopolysaccharide biosynthesis in enteric bacteria. *Microbiol Rev* **57**, 655–682 (1993).

Yethon, J. A., Vinogradov, E., Perry, M. B. & Whitfield, C. Mutation of the lipopolysaccharide core glycosyltransferase encoded by *waaG* destabilizes the outer membrane of *Escherichia coli* by interfering with core phosphorylation. *J Bacteriol* **182**, 5620–5623 (2000).
